# RanDepict: Random chemical structure depiction generator

**DOI:** 10.1186/s13321-022-00609-4

**Published:** 2022-06-06

**Authors:** Henning Otto Brinkhaus, Kohulan Rajan, Achim Zielesny, Christoph Steinbeck

**Affiliations:** 1 Institute for Inorganic and Analytical Chemistry, Friedrich-Schiller-University Jena, Lessingstr. 8, 07743 Jena, Germany; 2 Institute for Bioinformatics and Chemoinformatics, Westphalian University of Applied Sciences, August-Schmidt-Ring 10, D-45665 Recklinghausen, Germany

**Keywords:** CDK, Chemical image depiction, Depiction generator image augmentation, Indigo, RDKit, OCSR

## Abstract

The development of deep learning-based optical chemical structure recognition (OCSR) systems has led to a need for datasets of chemical structure depictions. The diversity of the features in the training data is an important factor for the generation of deep learning systems that generalise well and are not overfit to a specific type of input. In the case of chemical structure depictions, these features are defined by the depiction parameters such as bond length, line thickness, label font style and many others. Here we present RanDepict, a toolkit for the creation of diverse sets of chemical structure depictions. The diversity of the image features is generated by making use of all available depiction parameters in the depiction functionalities of the CDK, RDKit, and Indigo. Furthermore, there is the option to enhance and augment the image with features such as curved arrows, chemical labels around the structure, or other kinds of distortions. Using depiction feature fingerprints, RanDepict ensures diversely picked image features. Here, the depiction and augmentation features are summarised in binary vectors and the MaxMin algorithm is used to pick diverse samples out of all valid options. By making all resources described herein publicly available, we hope to contribute to the development of deep learning-based OCSR systems.

**Graphical Abstract:**

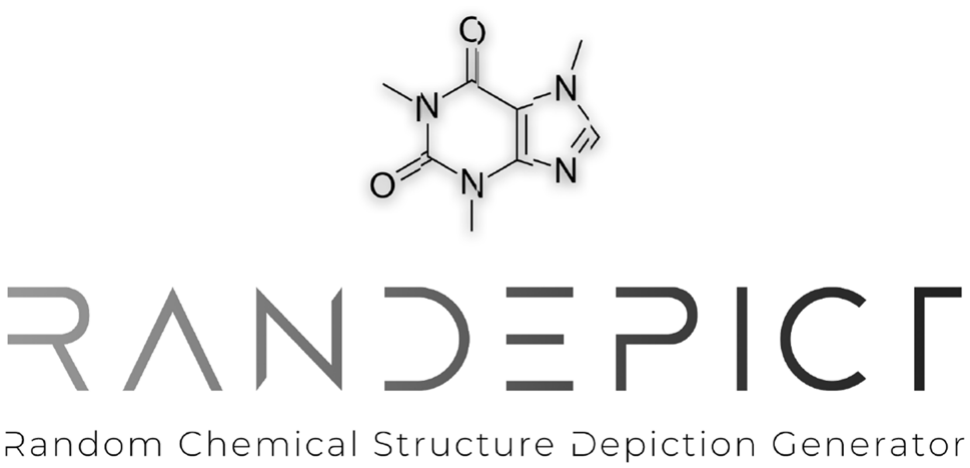

## Introduction

Since 2019, there has been a lot of development in the field of deep learning-based optical chemical structure recognition (OCSR) [1–7]. This indicates a paradigm shift as convolutional neural networks (CNN) as encoders in combination with recurrent neural networks (RNN) or transformers as decoders replace the rule-based systems that have previously defined the standard in the field [8].

The rule-based systems typically apply a workflow of binarisation, vectorisation, the detection of specific structural elements like dashed lines and wedges, optical character recognition (OCR), graph compilation and additional post-processing steps. Every single step in these workflows can be fine-tuned to achieve optimal results. In 2021, Clevert et al. have shown that the openly available rule-based systems surprisingly fail on the common benchmark datasets when slight image perturbations like rotation and shearing are introduced [3]. This lack of robustness is a clear indication that these systems have been overfitted to the benchmark datasets and that there is a need for more diverse benchmark data.

**Fig. 1:**
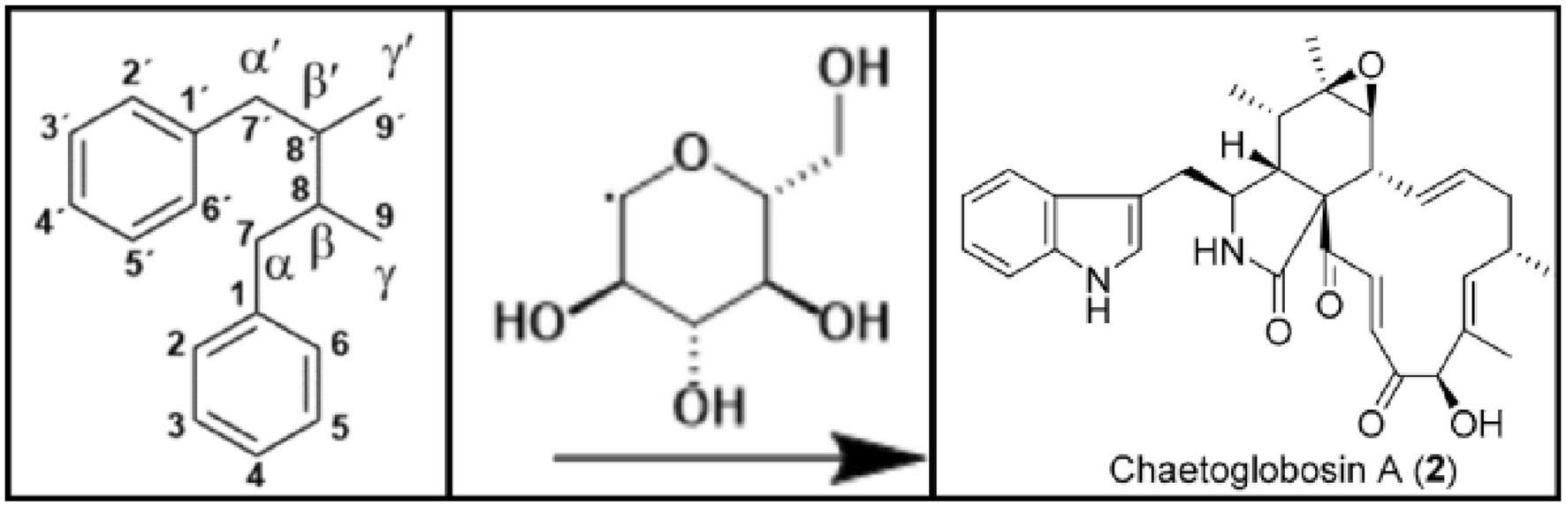
Examples of structure depictions from chemical publications extracted using DECIMER Segmentation which contain non-structural elements like atom labels (left) [11], reaction arrows (middle) [12] and identity labels (right) [13]

A machine-learning system learns to adapt its actions based on given environment information. Consequently, the quality of the environment information is a crucial factor for the system learning to solve a specific task [9]. Machine-learning systems are able to learn best when the input data they receive is similar to the data they have been trained on. In the case of most deep learning-based OCSR systems, the training data consists of images with depictions of chemical structures which are mapped to string representations of the underlying molecular graph. To be able to generalise well across a variety of different depiction styles, a machine-learning model needs to be trained on these depiction styles as well. Additionally, chemical structure depictions often contain non-structural elements like atom numbering or mechanism arrows which need to be considered as common noise types (Fig. 1). This is particularly relevant for real-world chemical data extraction applications, since the only openly available deep learning-based segmentation tool for chemical structures, DECIMER Segmentation, tends to include these non-structural elements in its output segments [10]. Hence, there is a need for a tool for the generation of chemical structure depictions of various depiction styles with additional non-structural elements.

We present RanDepict, a toolkit for generating diverse representations of chemical structures. It addresses the problem of the generation of diverse training data for OCSR tools by pseudo-randomly setting the available depiction parameters when depicting a structure with one out of three cheminformatics toolkits (Chemistry Development Kit (CDK) [14], RDKit [15] and Indigo [16]). Various augmentations such as image perturbations or non-structural elements like labels and curved arrows can also be added. Instead of pseudo-randomly picking depiction and augmentation parameters, there also is the option to generate the images based on depiction feature fingerprints. Here, the depiction and augmentation parameters are represented as bit arrays and RDKit’s implementation of the MaxMin algorithm [17] is used to pick diverse samples out of all valid fingerprints.

By making it publicly accessible, we hope to contribute to the development of robust deep learning-based OCSR systems by providing diverse training and benchmark datasets. RanDepict’s source code is publicly available on GitHub.

## Implementation

RanDepict is written using Python 3 [18]. The chemical structure depictions are generated using the CDK, RDKit and Indigo. As CDK is Java-based, its classes are accessed in Python via JPype [19].

When a chemical structure depiction is generated, one of the three above-mentioned cheminformatics toolkits is picked randomly. Then, the depiction functions arbitrarily define all available parameters. Among these parameters are bond length, thickness, style, kékulisation, font type and size of atom labels, rotation of molecules, the distance between lines and labels and the abbreviation of chemical substructures. Here, the abbreviation of chemical substructures means that, for example, a tertiary butyl group is abbreviated as tBu instead of drawing the full branched chain. Additionally, atom numbering and chirality labels are included in the depiction parameters as they are added by the cheminformatics toolkits and not by separate functions.

Various non-structural features can be added to the structure depiction. Along with atom numbering and chirality labels, there are also curved mechanism arrows, straight reaction arrows, chemical identity labels, rest group labels, and reaction condition labels.

The arrow images are randomly picked from a set of available images, resized, rotated, and pasted in a position where they do (curved arrows) or do not (reaction arrows) overlap with the chemical structure depiction.

The labels are generated by arbitrarily combining a variety of available text elements. For example, a chemical identity label is generated as a number (e.g., ‘1’), a number-letter combination (e.g., ‘1a’), a number-number combination (e.g., ‘1–4’) or a number-letter-letter combination (‘1a–d’). Similarly, rest group labels are generated by combining rest group variables (e.g., ‘R’, ‘X’) with randomly picked superatom labels. The list of superatoms that is used here was originally published along with the rule-based OCSR system OSRA [20]. Reaction condition labels are generated by combining the name of a chemical compound, a solvent, and a time. The font size and type for the labels are randomly chosen. The available font types include standard fonts like Arial and Times New Roman but also fewer common fonts that contain, for example, Asian or Greek-style characters. This ensures that there are diverse types of non-structural elements around the chemical structure that a potential deep learning-based OCSR system can learn to ignore as noise. Furthermore, the image augmentation library imgaug is used to add additional image perturbations. This includes a mild rotation, shearing, salt and pepper noise, brightness and colour adjustments, JPEG compression and pixelation.

Every image created by RanDepict with the desired shape of (m, n) is slightly distorted and resized. Therefore, it is first generated with a shape of (m_dist_, n_dist_) where m_dist_ and n_dist_ are randomly drawn from [0.9*m, 1.1*m] and [0.9*n, 1.1*n]. Then, it is resized to the desired shape (m, n) with a randomly picked resizing method. The purpose of this procedure is the introduction of the artefacts of different resizing methods in the image data.

Whenever a (pseudo-)random decision is made, the seed attribute of the RandomDepictor class is used as a seed for the pseudo-random choice and then altered systematically. This ensures that the creation of datasets with RanDepict is reproducible under the condition that the tool is fed the same SMILES input and the same initial seed.

**Fig. 2:**
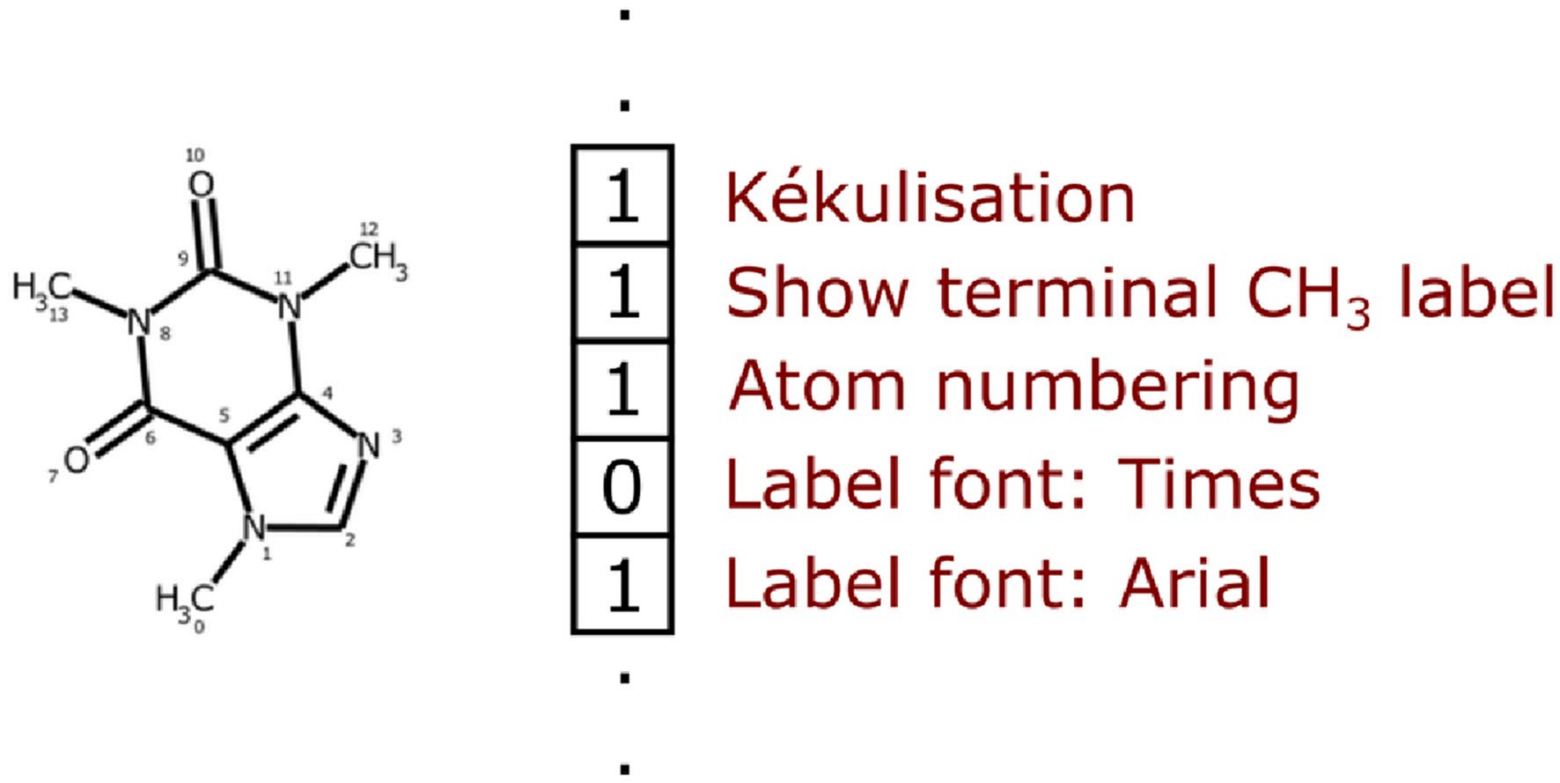
Exemplary illustration of depiction feature fingerprints

Since the entire depiction parameters constitute a high-dimensional feature space, random sampling does not necessarily guarantee even coverage. Instead of choosing parameters randomly, RanDepict can use depiction feature fingerprints to deal with this issue. This means that all depiction parameters as well as the presence or absence of the different augmentation types are summarised in bit arrays. Here, a 1 or a 0 in every position represents the presence or absence of a certain feature (exemplary illustration in Fig. 2). After computing all possible valid fingerprints, RDKit’s implementation of the MaxMin algorithm [17] is used to pick diverse samples. This way, diversity of depiction features is ensured.

The set of all possible valid fingerprints is determined as the combination of all valid fingerprint building blocks in a given order. Here, a fingerprint building block is a valid subset of values that are linked to certain positions in the whole fingerprint which express one depiction feature. A valid fingerprint is a combination of values that does not lead to contradicting statements about the underlying chemical structure depiction.

Let an exemplary chemical structure depiction be defined by the two features kékulisation and bond width. The kékulisation is defined on position 0 of the fingerprint. The resulting building block for this feature is (0, 1) as the first position of the fingerprint can take these two values to refer to whether the kékulisation is being applied or not. Assuming that the bond width can be thin, medium, or bold, these options would be described by positions 1–3 of the fingerprint. The building blocks for the feature bond width would be (1,0,0), (0,1,0) and (0,0,1). Other combinations for these positions would be invalid as, for example, the combination (1,0,1) on these fingerprint positions would refer to the bond width being thin and bold at the same time. The combination of the valid building blocks for all features in the given order defines the set of all fingerprint combinations. In the aforementioned example, this results in (0,1,0,0), (0,0,1,0), (0,0,0,1), (1,1,0,0), (1,0,1,0) and (1,0,0,1) as the set of valid fingerprints.

The building blocks of the fingerprints are generated automatically. A pseudo-random decision during the depiction creation just needs to be flagged as relevant for the fingerprint. RanDepict recognises this and automatically generates a fingerprint scheme. This way, the code for the fingerprint generation does not need to be adapted in the case of modifications in the depiction creation process.

During the fingerprint generation process, every binary decision (kékulisation in the example above) is simply allocated to one position in the bit array. When categorical decisions (bond width in the example above) are allocated to as many positions as there are categories where every position then indicates the presence or absence of a certain category and only one of them can have the value 1. Numerical ranges are split into three subranges which are then treated like categories. For example, if the bond width could be described by an integer with the possible values [1, 2, 3, 4, 5, 6] this would be allocated to three positions in the fingerprint. These positions would be linked to the subsets [1, 2], [3, 4] and [5, 6]. This means that the fingerprint does not always define an exact value for certain parameters but only specifies a range. When creating a depiction from a fingerprint, the parameter is randomly drawn from this subrange. This is necessary to reduce the number of possible fingerprints as the combinatorial explosion complicates computing all possible fingerprint combinations otherwise.

The three cheminformatics toolkits offer varying amounts of adjustable parameters. During the creation of a CDK depiction, 15 parameters are set. When using RDKit and Indigo, 10 and 8 parameters are adjustable. The ranges of possible values for these parameters differ between the tools. Hence, fingerprints for CDK, RDKit and Indigo depictions and the additional augmentations are four separate entities. The augmentation fingerprints only describe the presence or absence of an augmentation feature but do not comprise the specific parameters which are set. The varying parameter numbers and ranges lead to strongly differing numbers of valid depiction feature fingerprints: 2,799,360 for the CDK fingerprints, 18,432 for RDKit fingerprints, 864 for Indigo and 2048 for the augmentations. When generating a dataset from the fingerprints the user can specify the desired proportions of CDK, RDKit and Indigo depictions as well as the proportion of structures with added augmentations. They default to 55% (CDK), 30% (RDKit) and 15% (Indigo), 50% (augmented).

## Results

**Fig. 3:**
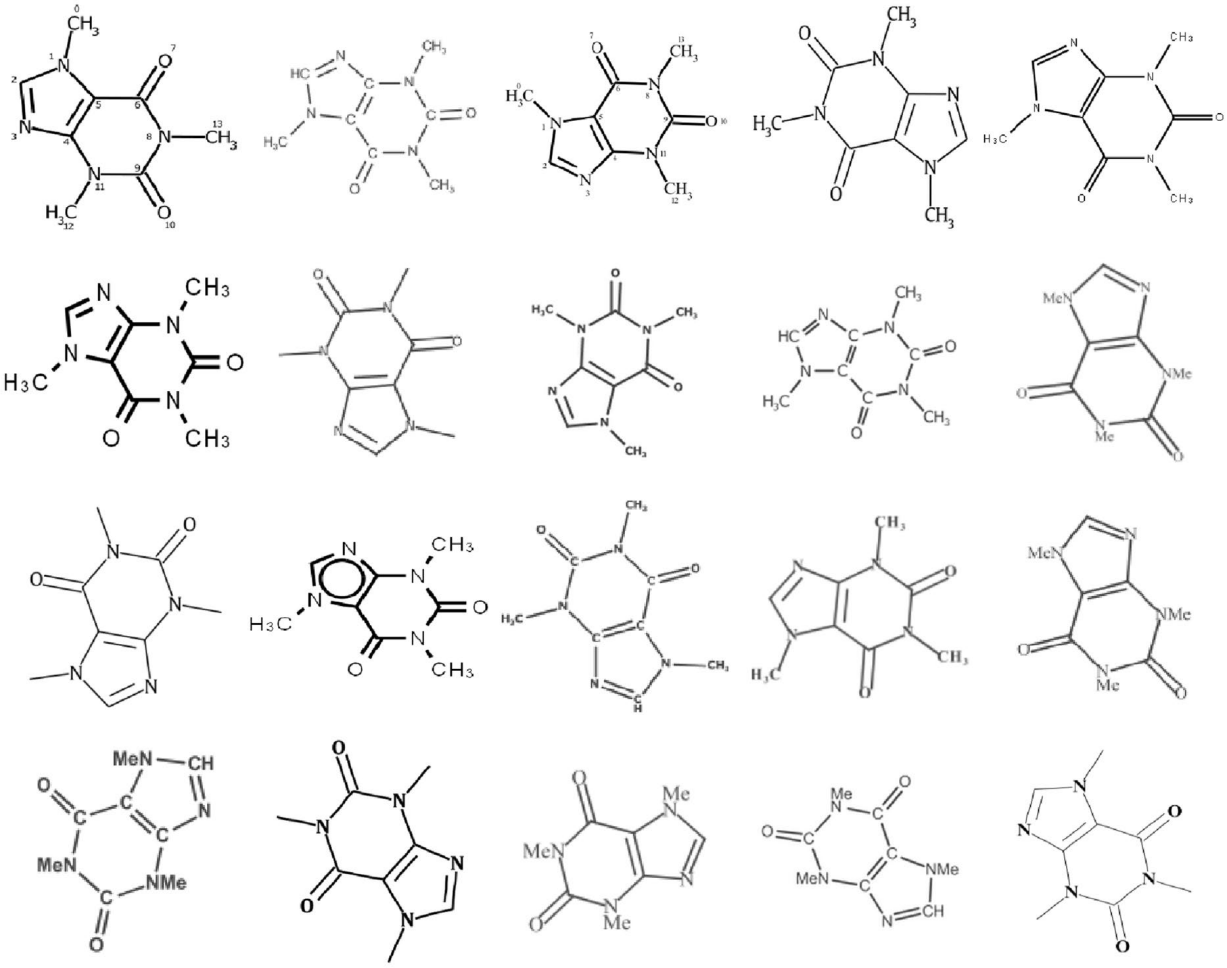
Depictions of caffeine with various depiction styles generated with RanDepict with feature fingerprint picking without additional augmentations

**Fig. 4:**
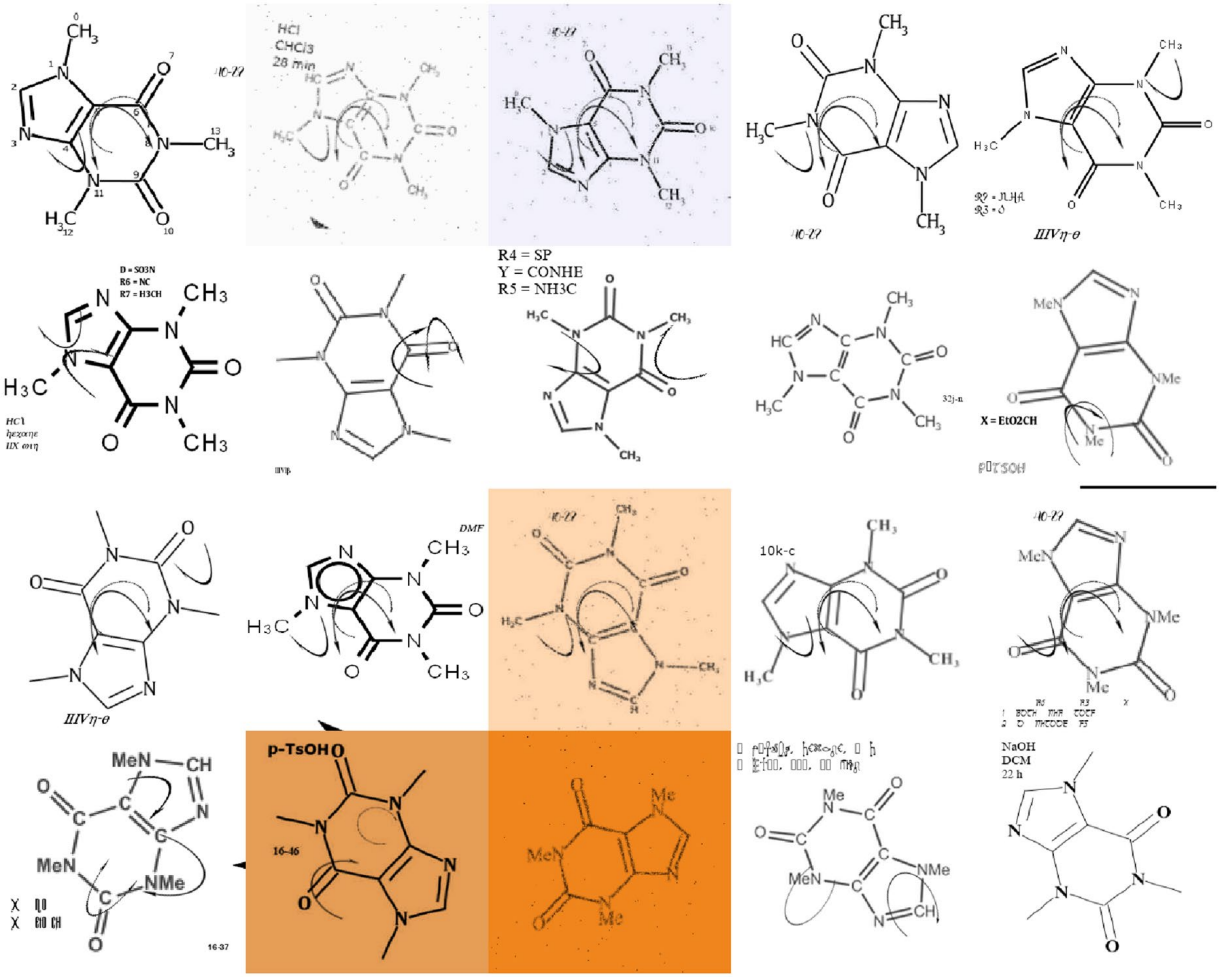
Depictions of caffeine with various depiction styles and additional non-structural features and noise types generated with RanDepict using feature fingerprint picking

RanDepict was designed to allow the generation of diverse chemical structure depictions using only a few lines of code. After generating a RandomDepictor object, the method random_depiction can be used to generate depictions of chemical structures. These depictions are generated by using randomly picked parameters in CDK, RDKit and Indigo without additional elements (Fig. 3). The object can be called as a function to generate chemical structure depictions with additional non-structural elements and augmentations (Fig. 4). There are various examples for the batch generation of structure depiction datasets with and without the usage of the feature fingerprint picking functionality in the documentation.

**Table:** 

from RanDepict import RandomDepictor
smiles = “CN1C = NC2 = C1C(= O)N(C(= O)N2C)C”
with RandomDepictor() as depictor:
# Generate chemical structure depictions
image = depictor.random_depiction(smiles)
# Generate augmented chemical structure depictions
augmented_image = depictor(smiles)

**Fig. 5:**
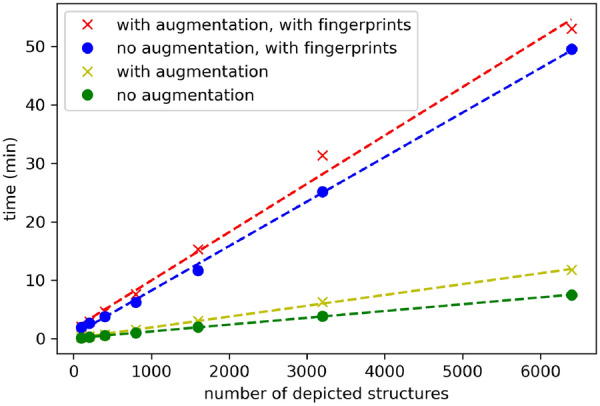
Runtime analysis of chemical structure depiction generation with RanDepict with and without augmentations and the application of the feature fingerprint picking functionality. The dotted lines represent linear regression results for each case

On a compute server with two Intel(R) Xeon(R) Silver 4114 CPUs and 64 GB of RAM, the runtime was evaluated for the generation of 100, 200, 400, 800, 1600, 3200 and 6400 chemical structure depictions with an image size of 299 × 299 (Fig. 5) using one CPU core. This was done with and without the addition of augmentations and the usage of the feature fingerprints. The linear regression results of the different runs clearly indicate that the runtime increases linearly with a growing amount of depictions.

Based on the regression analysis, the generation of one million chemical structure depictions without the feature fingerprints takes 19 h without augmentations and 31 h with augmentations. For the generation of large datasets consisting of millions of structures, it is recommended to split the input SMILES lists and run the generation in parallel on multiple nodes in a cluster or using a cloud service. As long as the initial seed is set differently in every parallel instance, different sets of parameters are picked.

The same extrapolation applied to the generation of one million structures using feature fingerprint selection results in 127 h without augmentations and 138 h with augmentations. The user could split up the input SMILES lists here, too, and initialise the MaxMin picking mechanism with different seeds on every instance in a computing cluster to ensure different sets of parameters are picked. Nevertheless, the creation of datasets from fingerprints is significantly slower than the generation with random parameter sampling. Depending on the desired dataset size, the user can decide whether to use depiction feature fingerprints. The feature fingerprint picking functionality is highly recommended for the generation of smaller test and benchmark sets as it ensures a diverse selection of features.

## Conclusions

RanDepict: a toolkit for generating chemical structure depictions. It features diverse structure depiction elements, as well as non-structural elements and image augmentations.

If desired, the diversity of depiction features is ensured by representing the entirety of features in bit arrays (feature fingerprints) and picking diverse sets using the MaxMin algorithm. Even though fingerprint picking is a time-consuming process, we highly recommend using it for the generation of smaller test sets where the random sampling of depiction features may not necessarily lead to a dataset that represents the entire feature space.

The complete source code of RanDepict, scripts for the generation of Figs. 3 and 4, the runtime determination as well as other examples for the usage and detailed documentation of RanDepict are openly accessible on GitHub and Read the Docs. It is possible to install RanDepict as a package via pip. We hope that our work will contribute to the standardisation of training and test datasets in the field of OCSR.

## Data Availability

Project name: RanDepict. Project home page: https://github.com/OBrink/RanDepict,  https://pypi.org/project/RanDepict/. Operating system(s): Linux, macOS and Windows 10. Programming language: Python 3. Other requirements: Python packages: numpy >= 1.19, imgaug, scikit-image, epam.indigo, jpype1, ipyplot, rdkit-pypi, imagecorruptions, pillow >= 8.2.0; Java Libraries: CDK 2.5. License: MIT. Any restrictions to use by non-academics: Not applicable.

## Abbreviations

CDK: Chemistry development kit
CNN: Convolutional neural network
JPEG: Joint Photographic Experts Group
OCSR: Optical chemical structure recognition
OSRA: Optical structure recognition application
RNN: Recurrent neural network
SMILES: Simplified molecular input line entry specification
